# Research landscape of genetics in dilated cardiomyopathy: insight from a bibliometric analysis

**DOI:** 10.3389/fcvm.2024.1362551

**Published:** 2024-07-12

**Authors:** Tiantian Chao, Yaru Ge, Jinghui Sun, Chenglong Wang

**Affiliations:** ^1^ National Clinical Research Center for Chinese Medicine Cardiology, Xiyuan Hospital, China Academy of Chinese Medical Sciences, Beijing, China; ^2^ Community Medical Center, Beijing Shijitan Hospital, Capital Medical University, Beijing, China

**Keywords:** dilated cardiomyopathy, genetics, bibliometric analysis, heart failure, sudden cardiac death

## Abstract

**Background:**

Dilated cardiomyopathy (DCM) is a heterogeneous myocardial disorder with diverse genetic or acquired origins. Notable advances have been achieved in discovering and understanding the genetics of DCM. This study aimed to depict the distribution of the main research forces, hotspots, and frontiers in the genetics of DCM, thus shaping future research directions.

**Methods:**

Based on the documents published in the Web of Science Core Collection database from 2013 to 2022, co-authorship of authors, institutions, and countries/regions, co-citation of references, and co-occurrence of keywords were conducted respectively to present the distribution of the leading research forces, research hotspots, and emerging trends in the genetics of DCM.

**Results:**

4,141 documents were included, and the annual publications have steadily increased. Seidman, Christine E, Meder, Benjamin, Sinagra, Gianfranco were the most productive authors, German Centre for Cardiovascular Research was the most productive institution, and the USA, China, and Germany were the most prolific countries. The co-occurrence of keywords has generated 8 clusters, including DCM, lamin a/c, heart failure, sudden cardiac death, hypertrophic cardiomyopathy, cardiac hypertrophy, arrhythmogenic cardiomyopathy, and next-generation sequencing. Frequent keywords with average publication time after 2019 mainly included arrhythmogenic cardiomyopathy, whole-exome sequencing, RBM 20, phenotype, risk stratification, precision medicine, genotype, and machine learning.

**Conclusion:**

The research landscape of genetics in DCM is continuously evolving. Deciphering the genetic profiles by next-generation sequencing and illustrating pathogenic mechanisms of gene variants, establishing innovative treatments for heart failure and improved risk stratification for SCD, uncovering the genetic overlaps between DCM and other inherited cardiomyopathies, as well as identifying genotype-phenotype correlations are the main research hotspots and frontiers in this field.

## 1 Introduction

Dilated cardiomyopathy (DCM) is characterized by enlarged left or biventricular diameters and impaired contractile performances in the absence of abnormal loading conditions or ischemic heart disease (1, 2). The broad spectrum of clinical presentations in DCM ranges from none to overt heart failure (HF), severe arrhythmia, thromboembolism, and sudden cardiac death (SCD) (2, 3). The estimated prevalence of DCM per 10,000 population in the UK was 4.3 cases, twice as common among men than women (4). Despite optimal medical and device therapy, the 5-year mortality rate in DCM remains as high as 15.5% (5). Two-thirds of affected patients die due to pump failure, followed by SCD in one-third of cases (6). DCM is an aetiologically heterogeneous myocardial disorder associated with genetic determinants interfering with environmental factors (7). Most common pathologies underlie reactive changes such as inflammation (viral myocarditis or autoimmune disease), nutritive-toxic influences (alcohol, drugs, chemotaxis), and metabolic disorders (1, 3). Substantial advances have recently been obtained in discovering and understanding the genetics of DCM, and the evidence base of pathogenic genes has been intensified either. Large-scale genomic sequencing has identified more than 100 genes associated with approximately 40%–50% of DCM patients, and these gene variants disrupt the function of multiple proteins involving cytoskeletal, sarcomere, or nuclear envelope, thus producing a final DCM phenotype (8). 12 genes (BAG3, DES, FLNC, LMNA, MYH7, PLN, RBM20, SCN5A, TNNC1, TNNT2, TTN) were evaluated as having definitive or strong evidence for DCM (9). Specific gene mutations may be particularly arrhythmogenic and associated with increased risk for SCD. For example, mutations in LMNA genes are associated with higher risks of SCD attributed to conduction disturbances (10). In addition, TTN, RBM20, BAG3, and LMNA significantly increased the genetic predisposition of HF (11). In a phenotypic clustering study of DCM using principal components analysis (12), 4 clinical distinct phenogroups were detected in DCM: (1) mild systolic dysfunction, (2) auto-immune, (3) genetic and arrhythmias and (4) severe systolic dysfunction. RNA-sequencing further revealed a distinct underlying molecular profile per group: pro-inflammatory, pro-fibrotic, and metabolic gene expression for clusters 2, 3, and 4 separately. Expanding understanding of the genetic architecture and the complex genotype-phenotype correlations of DCM has great implications for early diagnosis, prognostic stratification, and targeted therapy.

Until now, documents that quantitatively illustrate the knowledge mapping of genetics in DCM have not emerged yet. Bibliometric analysis is a practical and professional tool to summarize and visualize large quantities of scientific data, thus presenting the intellectual structure and academic trends of a research topic (13, 14). Employing CiteSpace and VOSviewer software, we mapped the global research landscape of genetics in DCM based on the documents from 2013 to 2022, aiming to depict the distribution of research hotspots and frontiers in this field to shape future research directions and provide references for policy development.

## 2 Materials and methods

### 2.1 Data source and retrieval strategy

The data source of this bibliometric analysis was the Science Citation Index Expanded (SCI-E) in the Web of Science (WoS) Core Collection database. Retrieval strategy: Topic Search = (gene* OR genetic* OR genome OR genomic*) AND Topic Search = (dilated cardiomyopathy). Only articles or reviews published in English from 1 January 2013 to 31 December 2022 were selected. Exclusion criteria: letter, news, proceeding paper, editorial material, meeting abstract, book chapter, retracted publication, publications with expression of concern, correction, and early access. The literature search was completed on 23 July 2023 to reduce the information bias caused by database updates. Tiantian Chao and Yaru Ge independently conducted the retrieval, and a third reviewer was consulted for inconsistencies. The retrieved documents were exported in plain text format as “full records and cited references” data, which included title, publication year, author, institution, country, number of citations, journals, keywords, references, and other information. CiteSpace 6.1.R2 software was used to remove duplicates.

### 2.2 Data analyses

Citespace 6.2.R2 and VOSviewer software were used to perform the bibliometric analysis through co-authorship, co-citation, and co-occurrence analyses. While co-authorship analyses were to measure collaborations of research teams, co-citation analyses to measure the influences of items, and co-word analyses to find connections among concepts that co-occur in documents (15). Normalization of keywords was conducted by merging synonyms, correcting spelling differences, and replacing abbreviated terms with full terms. The top 50 most cited or occurred items from each time slice were set as the node selection criteria in Citespace. In the network mapped by Citespace, nodes with betweenness centrality ≥0.1, characterized by purple halos in the circle, are often at the convergence point of different clustering paths. In other words, it has the role of “bridge” and is often called a turning point (16).

Co-authorship analyses of authors, institutions, and countries/regions were conducted to distribute the main research forces in this field. Highly co-cited references were identified to present the knowledge bases in this area. While co-occurrence analyses of keywords were conducted to display the research hotspots and emerging trends in the genetics of DCM.

## 3 Results

### 3.1 The annual number of publications

**Figure 1:**
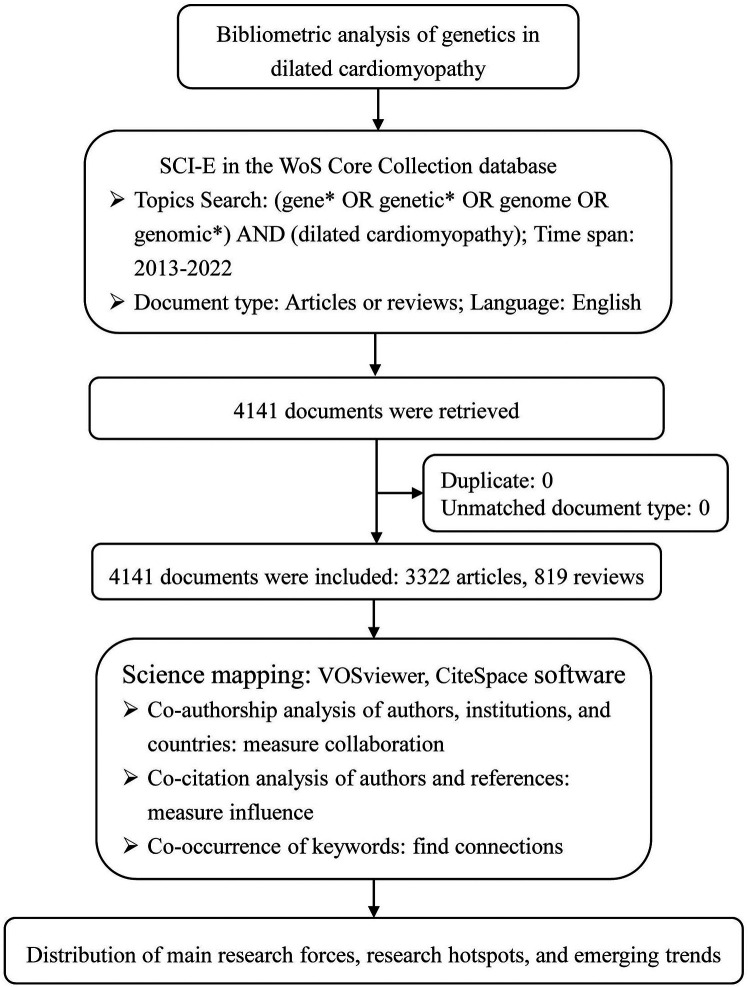
Flow chart of bibliometric analysis.

**Figure 2:**
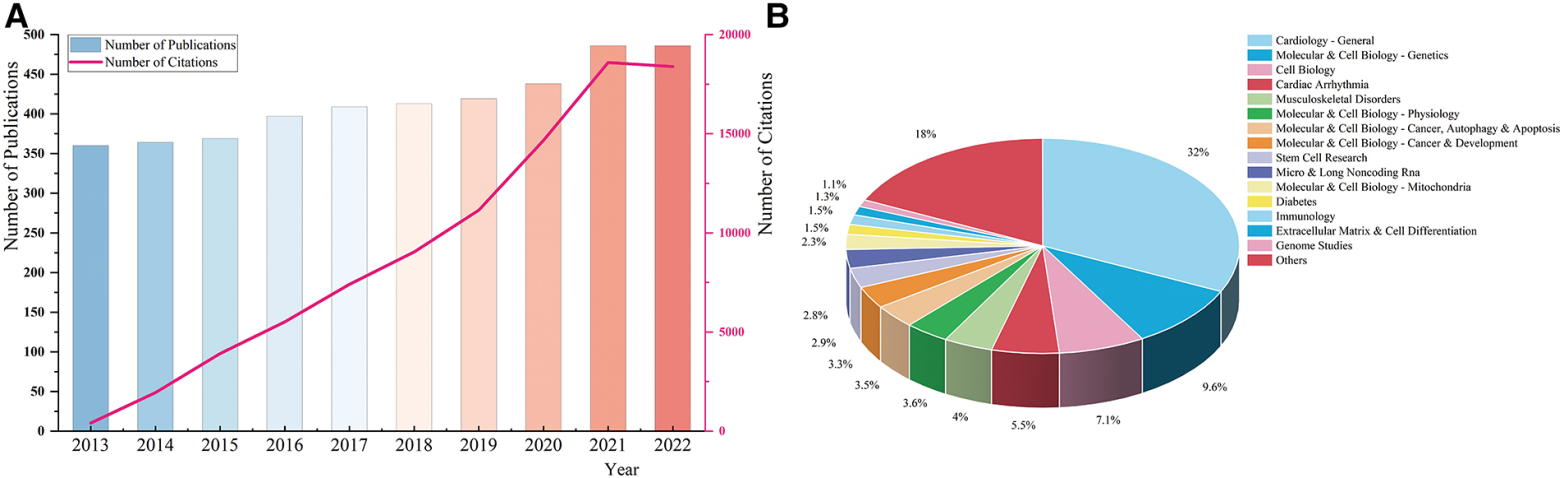
(**A**) The annual number of publications and citations. (**B**) The distribution of macroscopic citation topics in the genetics of dilated cardiomyopathy.

4,141 documents were retrieved and enrolled in this study, including 3,322 articles and 819 reviews (Figure 1). As (Figure 2A) showed, the annual number of publications has increased steadily, with 360 documents in 2013 and 486 papers in 2022, while two relatively rapid growth trends existed in 2015 and 2020. The annual number of citations has also demonstrated a fast-growing trend. This indicates that genetics has always been one of the most active research hotspots in DCM, and in the years to come, researchers’ interest in it seems unlikely to fade either.

As (Figure 2B) demonstrated, cardiology-general, molecular & cell biology-genetics, cell biology, cardiac arrhythmia, and musculoskeletal disorders were the main citation topics in the research field of genetics in DCM.

### 3.2 Authors and co-cited authors

**Figure 3:**
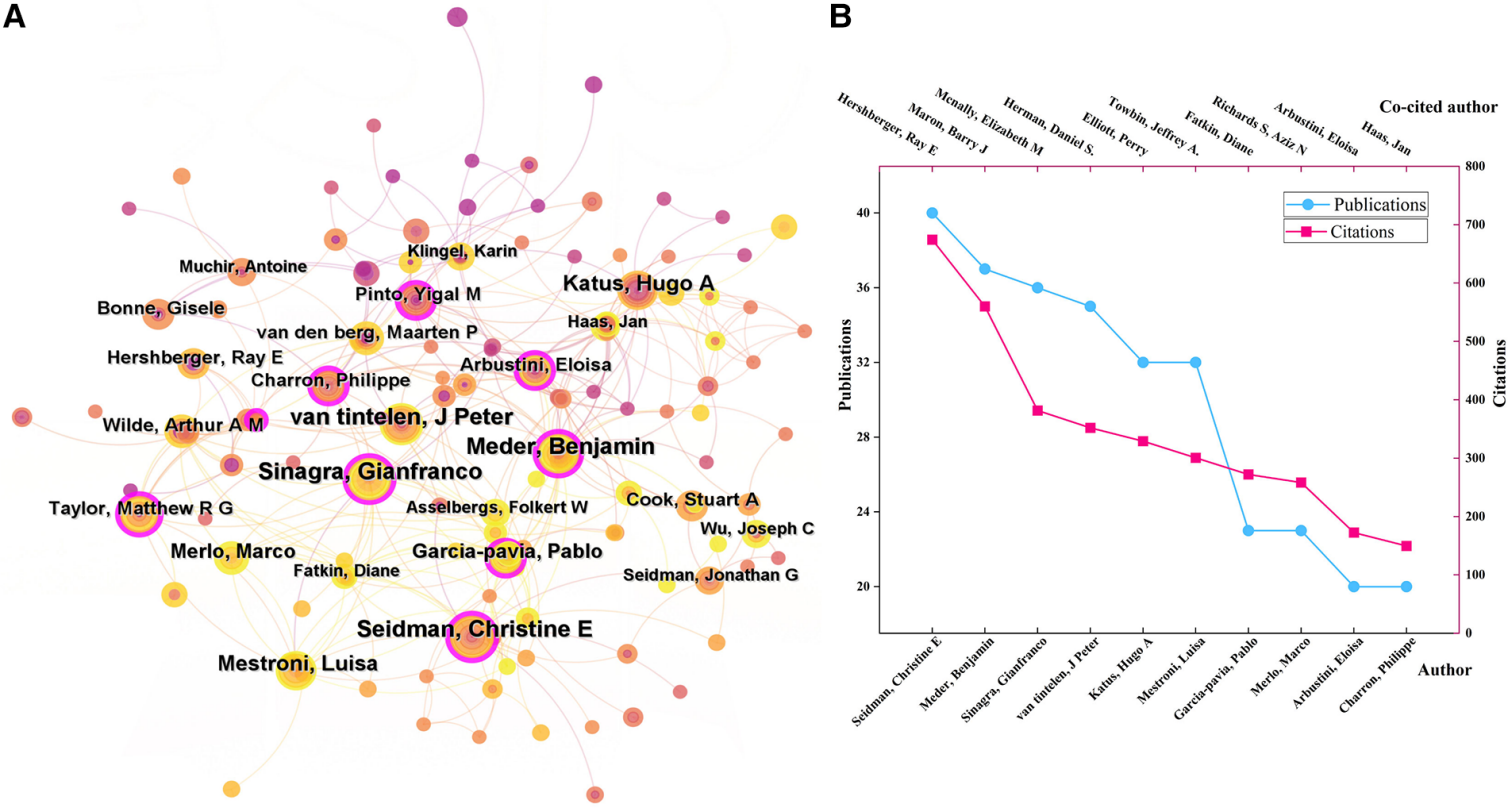
(**A**) The author collaboration networks. (**B**) The top 10 authors with the highest publications and citations.

There were 31 authors who all published more than ten documents. As was shown in (Figure 3A), Seidman, Christine E (USA, 40 papers), Meder, Benjamin (Germany, 37), and Sinagra, Gianfranco (Germany, 36) were the top 3 most productive authors in this field. They also had high betweenness centrality, while Arbustini, Eloisa in Italy had the highest betweenness centrality (0.18), indicating that they were key contributors in this field, functioning as bridges to strength contacts among other authors.

Author co-citation analysis means two or more authors are simultaneously cited in a document. The high citation frequencies reflect the significant influence of scholars on the research field. There were 42 authors with co-citation frequencies more than 100 times. As (Figure 3B) demonstrated, the top 5 most co-cited authors were Hershberger, Ray E (690 citations), Maron, Barry J (590), Mcnally, Elizabeth M (434), Herman, Daniel S. (408), and Elliott, Perry (388).

### 3.3 Institutions

**Figure 4:**
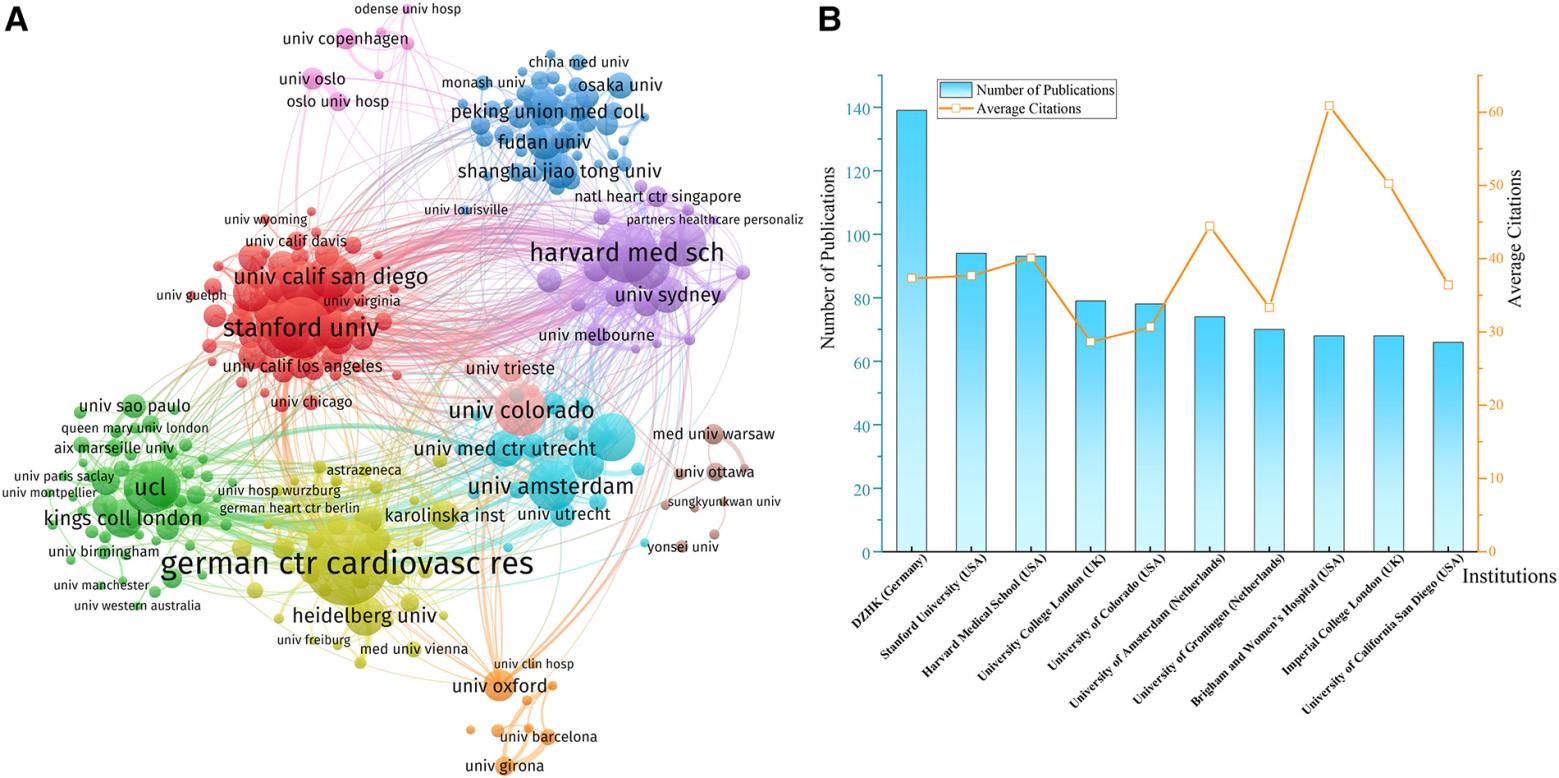
(**A**) Network of institution collaborations. (**B**) The top 10 most productive institutions. DZHK, German centre for cardiovascular research.

Institutions that have published at least ten documents were included in the network. German Centre for Cardiovascular Research (DZHK) was the most productive institution with 139 papers, followed by Stanford University (94), Harvard Medical School (93), University College London (UCL, 79), and University of Colorado (78). DZHK and Harvard Medical School cooperated most frequently with other institutions, and their total cooperation strengths were also the highest (Figure 4A). Although UCL, Imperial College London, and Stanford University also established broad collaborations with many other institutions, the cooperation between them was relatively short-term and unstable. (Figure 4B) showed that Brigham and Women's Hospital and Imperial College London had the highest average citations, which suggested the huge academic influences of their research achievement.

### 3.4 Countries

**Figure 5:**
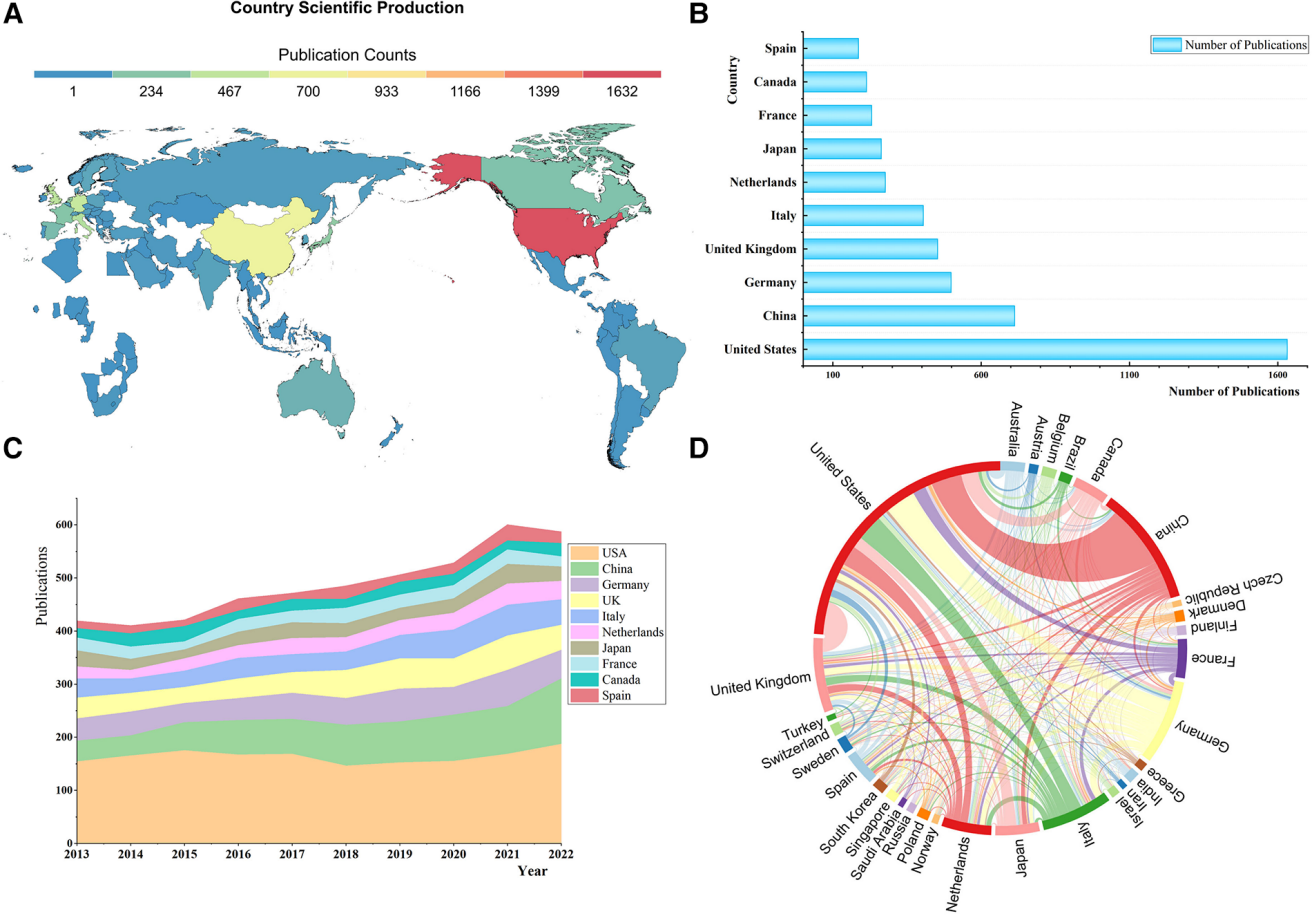
(**A)** World map of country scientific production. (**B**) The top 10 most productive countries. (**C**) The stacked area chart of annual publications in countries. (**D**) National collaboration map.

The USA (1,632), China (713), Germany (499), the UK (453), and Italy (405) were the top 5 most prolific countries in this field (Figures 5A,B). As (Figure 5C) shows, only the USA and China have maintained a growth trend in publications since 2020, while other countries’ scientific production all decreased to different degrees. The USA was prominently positioned in the international collaboration network, and it had partnerships with 29 countries worldwide, with China, Germany, Italy, and the UK being the closest (Figure 5D). China has established collaborations with 23 countries, while Sino-American cooperation was the most critical component of China's territorial cooperation.

### 3.5 Co-cited references

The cluster analysis of co-cited references and its timeline diagram can show this research field's knowledge bases and evolution process. The clustering labels are extracted from keywords of citing papers by the Log-Likeliood Ratio algorithm.

**Figure 6:**
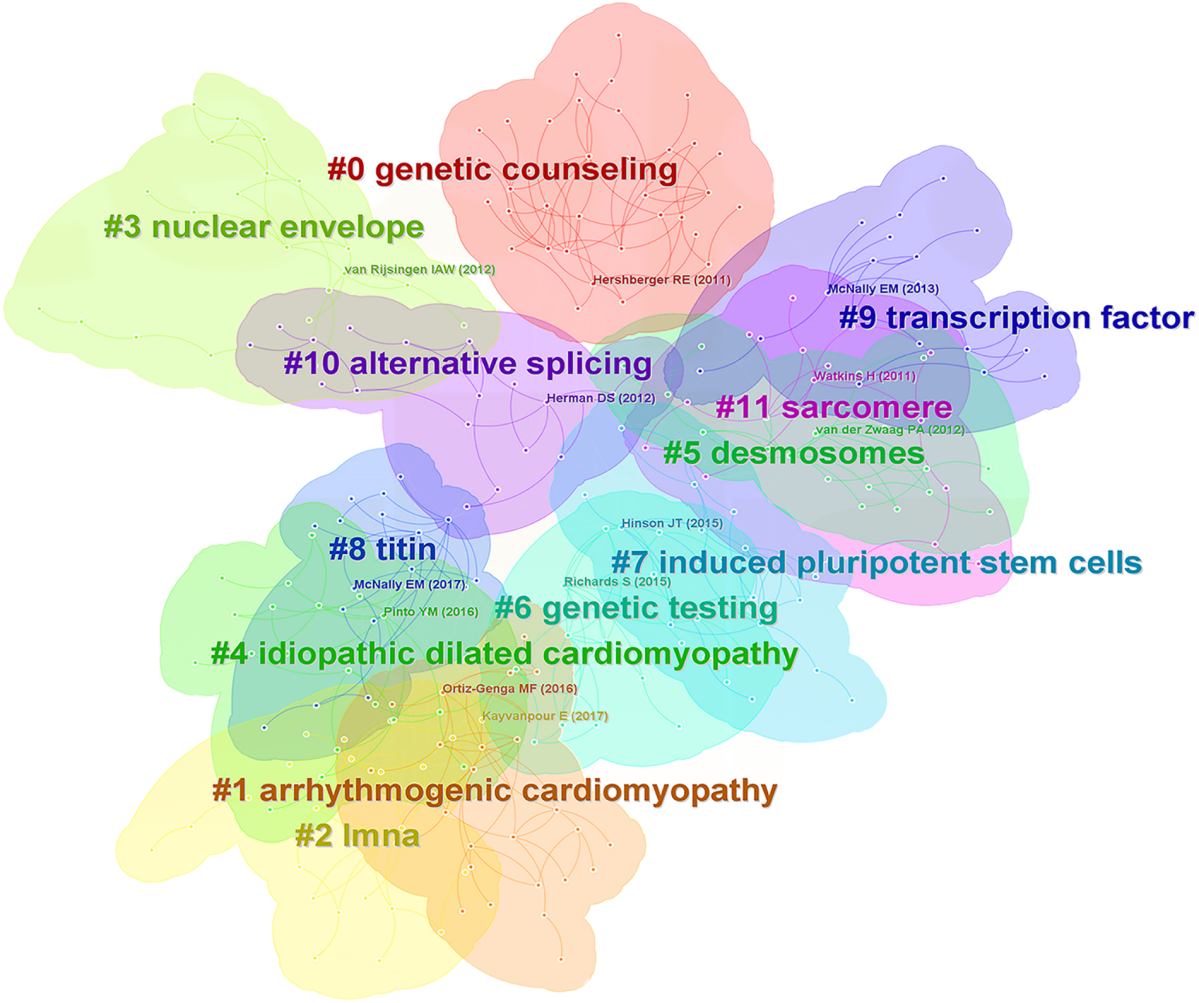
Cluster analysis of co-cited references.

Among the top 10 most cited references, five reviews presented the landscape of genetic mutations in DCM (3, 17–20), 2 articles explored the pathogenic mechanisms of TTN truncating variants (21, 22), three guidelines were related interpretation of sequence variants (23), updated definition of DCM (24), and treatment of HF (25). Figure 6 demonstrated that cluster analysis of co-cited references has generated 12 large clusters: #0 genetic counseling, #1 arrhythmogenic cardiomyopathy, #2 LMNA, #3 nuclear envelope, #4 idiopathic dilated cardiomyopathy, #5 desmosomes, #6 genetic testing, #7 induced pluripotent stem cells, #8 titin, #9 transcription factor, #10 alternative splicing, #11 sarcomere. Partial clusters are interrelated and can be further grouped. Lamin A/C is a nuclear envelope protein encoded by the LMNA gene through variable splicing. LMNA mutations classically cause both DCM and progressive conduction disease (26). Titin, coding by the TTN gene, is the largest sarcomeric protein within the myocardium, and TTN truncating variants (TTNtv) are present in 20%–25% of familial DCM (24). Mutations in desmosomal protein genes most commonly cause arrhythmogenic cardiomyopathy (ACM) (27). While desmosomal variants also underlie the genetic basis of DCM. Of note, genetic testing can be helpful in combination with genetic counseling for patients with inherited cardiomyopathy, which can help confirm the diagnosis and facilitate familial cascade screening (28). Somatic cells isolated from patients with inherited cardiomyopathy can be reprogrammed into induced pluripotent stem cells (iPSCs), subsequently differentiated into cardiovascular cell types. IPSCs-derived cardiomyocyte-based disease model was exploited to study the underlying pathogenic mechanisms of DCM (29). Alternative splicing is crucial for gene expression and proteome diversity, which enables genes to generate a diverse array of mature mRNA transcripts that can be translated into functionally different proteins (30).

### 3.6 Co-occurrences of keywords

**Figure 7:**
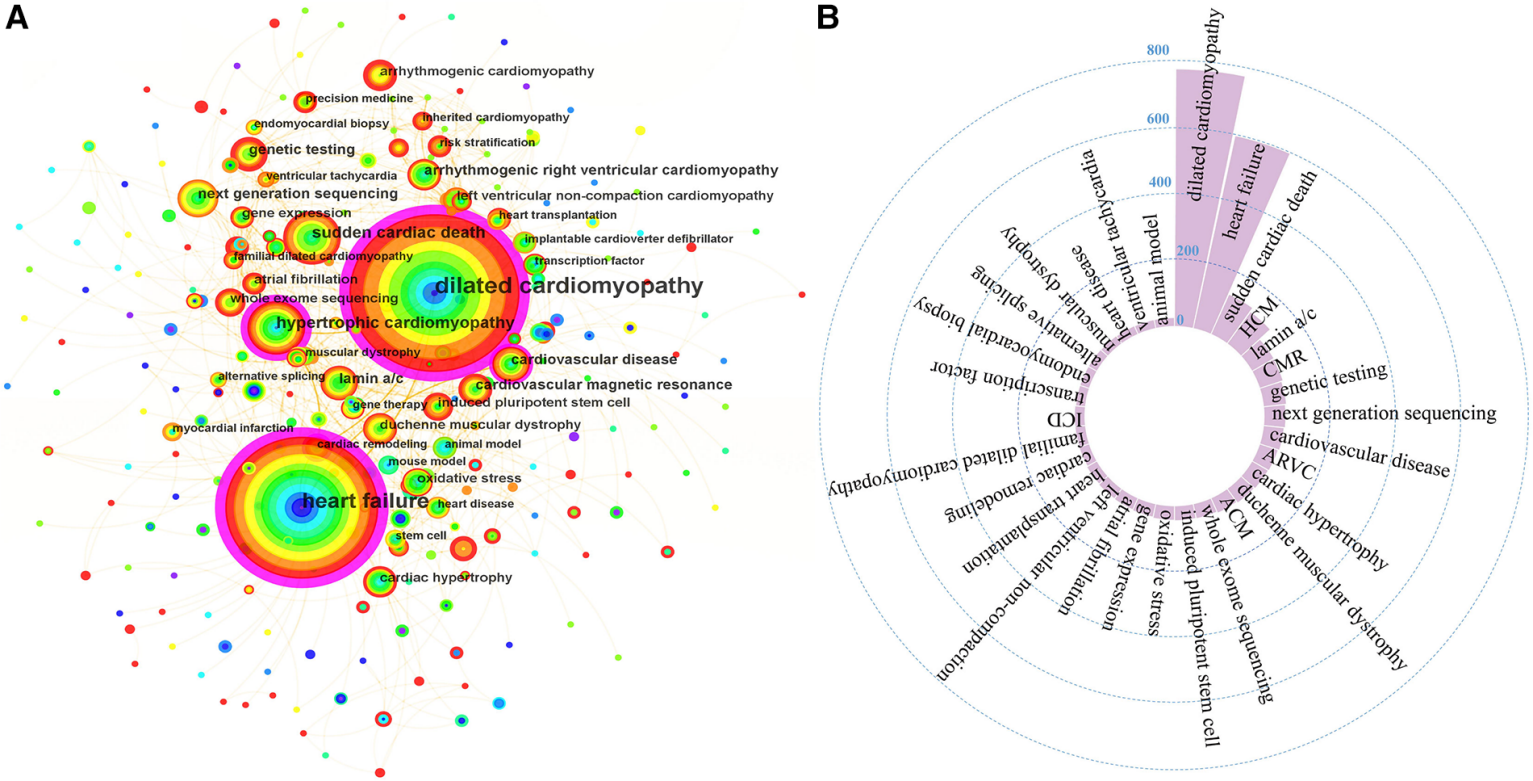
(**A**) The co-occurrence network of keywords. (**B**) High frequent keywords. ACM, arrhythmogenic cardiomyopathy; ARVC, arrhythmogenic right ventricular cardiomyopathy; CMR, cardiovascular magnetic resonance; HCM, hypertrophic cardiomyopathy; ICD, implantable cardioverter defibrillator.

**Figure 8:**
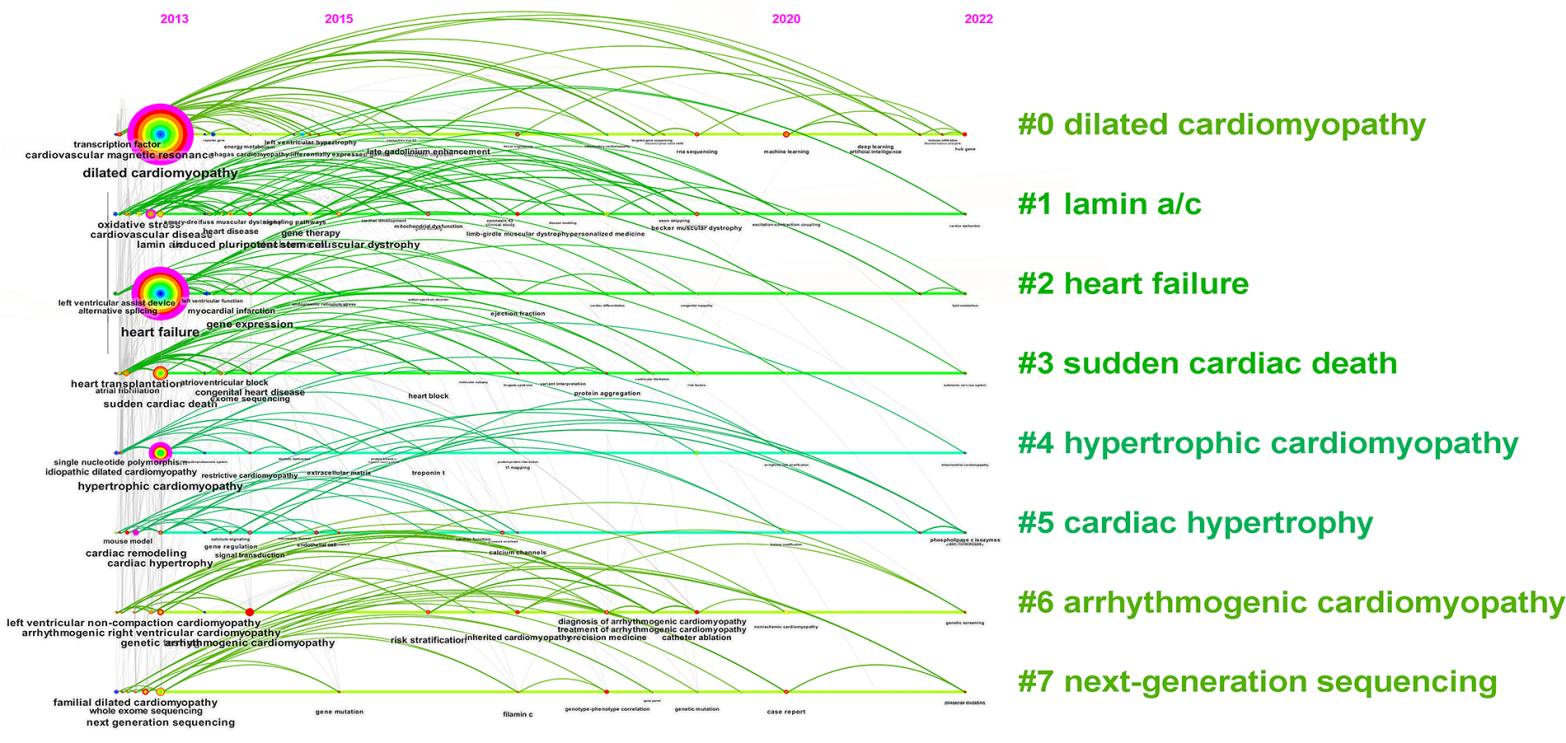
The timeline view of keywords.

Keywords are summarized expressions of the core contents of documents. The co-occurrence of keywords can reflect the research hotspots and thematic evolution of the research field. Detailed keywords normalization process was shown in the Supplementary Material. The size of nodes is parallel to the occurrence frequency of keywords, and the lines represent the co-occurrence relationship between keywords. The top 10 most frequent Keywords were DCM, HF, SCD, hypertrophic cardiomyopathy (HCM), lamin a/c, cardiovascular magnetic resonance (CMR), genetic testing, next-generation sequencing (NGS), cardiovascular disease, and arrhythmogenic right ventricular cardiomyopathy (Figures 7A,B). While DCM, HF, HCM, CMR, cardiovascular disease, and cardiac remodeling also had high betweenness centralities (Figure 7A). Cluster analysis of keywords generated eight large clusters (Figure 8), including #0 dilated cardiomyopathy, #1 lamin a/c, #2 heart failure, #3sudden cardiac death, #4hypertrophic cardiomyopathy, #5 cardiac hypertrophy, #6 arrhythmogenic cardiomyopathy, #7 next-generation sequencing. keywords within one cluster is on the same horizontal line, and the publication time is at the top of the image. The timeline view presents a subject’s rise, boom, and decline.

**Figure 9:**
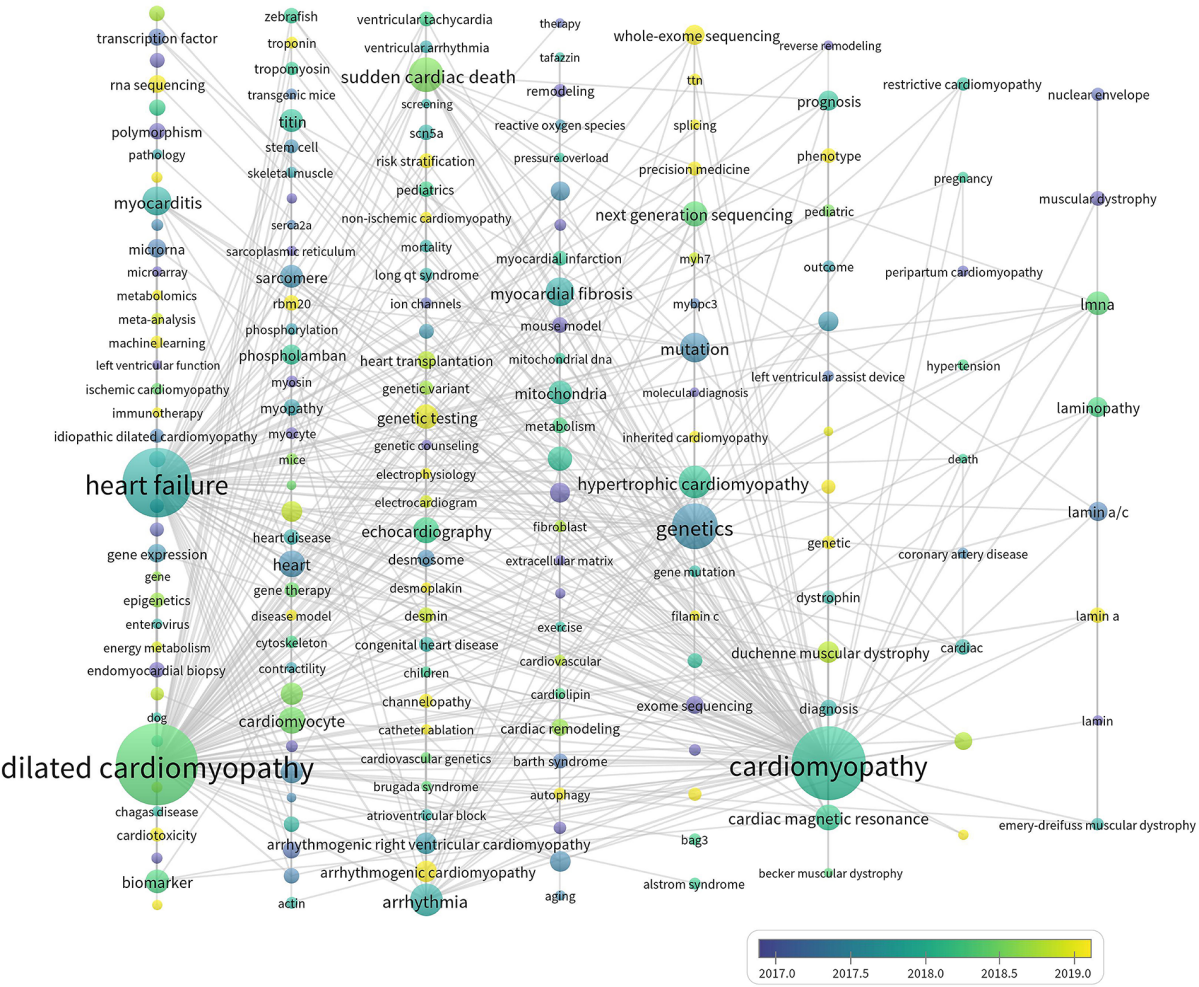
The thematic evolution of keywords.

As (Figure 9) showed, keywords with average publication time after 2019 and relatively high occurrence frequencies included ACM, whole-exome sequencing, RNA sequencing, RBM 20, phenotype, risk stratification, precision medicine, genotype, machine learning, and autophagy. Undoubtedly, these directions are the emerging trends in the research field of genetics in DCM.

## 4 Discussion

### 4.1 General information

A steadily growth trend of annual publications and citations from 2013 to 2022 suggested the researcher’s persistent enthusiasm and unremitting efforts in this field. Christine E. Seidman was the most productive researcher in this field, serving in the Cardiovascular Genetics Center at Brigham and Women's Hospital and Harvard Medical School. Her main research direction concerns cardiovascular disease-causing gene mutations and genetic variations that increase disease risk. DZHK, Stanford University, and Harvard Medical School held leading positions in scientific output. While the whole institutional collaboration network presented a globally dispersed and locally focused scene, thus international multi-center cooperations should be strengthed in the future. Regarding national contributions, the USA, China, and Germany were far ahead. The USA was also the national collaboration center. Although China ranked second in national publications, the relatively low collaboration intensity implied that Chinese researchers should deepen cooperation with institutions abroad.

### 4.2 Research hotspots and emerging trends

#### 4.2.1 Genetic testing and next-generation sequencing

Genetic testing is recommended to determine whether a pathogenic variant can be discovered to promote patient management and cascade screening (31). NGS offers unprecedented possibilities for discovering novel gene variants and expanding genetic testing panels (32). By employing massively parallel DNA sequencing technologies, NGS can sequence several dozen genes simultaneously, cost-effectively, and accurately (33). NGS has been applied to sequence the whole human genome (coding and non-coding regions of DNA) and the exome (coding regions of the genome) (8), significantly promoting our understanding of the monogenic causes of DCM.

HRS/EHRA expert consensus statement recommended that DCM patients with significant cardiac conduction disorder and/or a family history of premature unexpected SCD should undergo comprehensive or targeted (LMNA and SCN5A) genetic testing (31). Genotyping generally begins with the proband or the most severely affected family member with the youngest onset. Diagnostic genetic testing is to identify the underlying pathogenic variant in an individual with definite or suspected cardiomyopathy (34). Predictive genetic testing in asymptomatic relatives aims to determine which members have inherited the family's causal variant and are at risk for disease development (33). While prognostic testing in a patient with obvious cardiomyopathy helps stratify a patient's prognosis by better stratifying patients at high or low risk of cardiac death (28). The American College of Medical Genetics standards has classified genetic variants into five categories: pathogenic, likely pathogenic, uncertain significance, likely benign, and benign (23).

#### 4.2.2 Genetic basis of DCM

DCM genetic risk primarily follows a classic autosomal dominant pattern of inheritance (35). Mutations in genes encoding sarcomere, cytoskeleton, nuclear envelope, desmosome, mitochondria, and ion channels have been implicated in DCM (18). Sarcomeres are the basic unit of contraction in cardiac muscle and are vital for force-generating. Mutations within genes encoding sarcomeric proteins, including titin (TTN), myosin heavy chain alpha and beta (MYH6, MYH7), tropomyosin 1 (TPM1), myosin-binding protein C3 (MYBPC3), troponin-C, troponin-I, and troponin-T (TNNC1, TNNI3, and TNNT2), cardiac actinin 1 (ACTN1) are directly linked to disordered force generation, thus leading to myocardial dysfunction and DCM (36). Mutations involving cytoskeletal proteins, like filamin C (FLNC), dystrophin (DMD), and vinculin (VCL), are susceptible to DCM phenotype by causing defects of force transmission (18). Desmosomes link the sarcolemma to cytoskeletal intermediate filaments. Mutations in desmosomal proteins, desmocolin 2 (DSC2), desmoglein 2 (DSG2), desmoplakin (DSP), and plakophilin 2 (PKP2), can lead to disruption of intercellular junctions and cardiomyocyte detachment. Desmosomal gene variants are commonly associated with ACM but can also cause DCM (37). Mutations in nuclear envelope intermediate lamin A/C filament protein, LMNA, are also identified as the second prevalent genetic cause of DCM. Besides the genetic mutations mentioned above, BAG3, RBM20, SCN5A, and PLN are common DCM-causing genes (1). An international panel of experts on DCM genetics evaluated published evidence on 51 genes relevant to DCM, and they summarized that 12 genes (BAG3, DES, FLNC, LMNA, MYH7, PLN, RBM20, SCN5A, TNNC1, TNNT2, TTN) had definitive or strong evidence, and 7 genes (ACTC1, ACTN2, JPH2, NEXN, TNNI3, TPM1, VCL) had moderate evidence (9).

#### 4.2.3 Pathogenic mechanisms of TTN and LMNA in DCM

Full-length titin is the largest protein in the human proteome. It extends half a sarcomere, with the N-terminus anchoring in the Z-disc and the C-terminus in the M-line. Titin comprises 364 exons that undergo extensive alternative splicing to produce many isoforms (N2BA, N2B, and short novex isoforms) (38, 39). TTNtv is now considered the most frequent monogenetic cause of DCM, underlying 15%–25% of cases of nonischemic DCM (38). In recent years, parameters that determine TTNtv pathogenicity and the molecular mechanisms of TTNtv causing DCM have been extensively explored.

Roberts et al. found that isoform, exon usage, and variant position were significantly associated with the pathogenicity of TTNtv. Frameshift, nonsense, and canonical splice site TTNtv are rich in DCM patients, Variants that truncate both principal isoforms (N2BA and N2B) of TTN and/or close to the C terminus, particularly can cause DCM with severe phenotypes (22). There are mainly two mechanisms involving the pathogenicity of TTNtv in DCM. Firstly, transcripts containing a protein-truncating variant undergo nonsense-mediated mRNA decay, leading to a reduced protein dose (haploinsufficiency). Secondly, the protein produced is loss of function or deleterious (poison-peptide) (38). In 2021, McAfee et al. first discovered that truncated titin proteins were present and relatively abundant in hearts from TTNtv DCM patients (40), and they also provide evidence of decreased quantities of full-length titin protein in TTNtv DCM hearts, which advocate both haploinsufficiency and poison peptide mechanisms. Schafer et al. discovered that distal I-band and all A-band TTNtv have higher risks than variants in other titin domains (41). Robert Romano et al. further demonstrated the position-dependent pathogenetic effect of TTNtv (42). An A-band TTNtv impaired sarcomere function more severely than an I-band TTNtv. Both A-band and I-band TTNtvs contributed to TTN haploinsufficiency, while only the A-band TTNtv generated abundant truncation titin proteins that damaged the structure and function of myofibrils.

Lamin A/C are nuclear envelope proteins encoded by the LMNA gene (26), the second most frequently implicated gene in DCM. Apart from maintaining nuclear structural stability, Lamins regulate gene transcription, chromatin organization, DNA replication, cytoskeletal/nucleus coupling, and signal transduction (43). LMNA mutations can cause diverse phenotypes, including DCM, Emery-Dreifuss muscular dystrophy, limb-girdle muscular dystrophy, lipodystrophy, progeria syndrome, and restrictive dermopathy (44). Hasselberg et al. found a prevalence of 6.2% for LMNA mutation in familial DCM, and young asymptomatic LMNA genotype-positive family members had high cardiac penetrance, 32% had atrioventricular block, 23% atrial fibrillation, and 39% non-sustained ventricular tachycardia (VT) (45). Whole-exome sequencing data suggested that the rare loss-of-function and missense LMNA variant was significantly related to atrial fibrillation, ventricular arrhythmias (VA), DCM, and HF (46). The transgenetic mice model partially revealed the molecular pathogenesis of LMNA mutations. Cardiomyocyte-specific expression of LMNA led to activation of the E2F/DNA damage response/TP53 pathway and induction of myocardial fibrosis, apoptosis, cardiac dysfunction, and premature death (47).

#### 4.2.4 Genotype-phenotype correlations

Gene variants perturb the biological function of multiple critical myocardial proteins and thus give rise to a final DCM phenotype (18). Generally, DCM patients with positive genetic testing results showed unfavorable prognoses than genotype-negative individuals (48). DCM patients with mutations in LMNA, PLN, RBM20, FLNC, DES, or SCN5A are associated with typical cardiac rhythm disorders ranging from atrioventricular blocks to supraventricular and malignant ventricular arrhythmias (MVA) (7). In comparison, desmosomal and LMNA variants had the highest rate of life-threatening arrhythmias regardless of the LVEF (49). This may lower the threshold for prophylactic ICD therapy and encourage enhanced rhythm surveillance. TTNtv patients developed with DCM at a higher age than LMNA subjects, less often developed severe left ventricular systolic dysfunction, had optimal medical responses, higher rates of left ventricular reverse remodeling (LVRR), and better composite outcomes than LMNA (50, 51). However, some studies highlighted that TTNtv could be an independent risk factor for arrhythmic events in DCM patients (52, 53). MYH7-related DCM is featured by early onset, high penetrance, and low LVRR. In addition, it has a lower incidence of end-stage HF and MVA than LMNA-related DCM and a similar risk to DCM caused by TTNtv (54). In recent years, data mining methods, such as machine learning, unsupervised clustering, and principal component analysis, have been applied to reclassify distinct DCM subgroups from clinical and imaging data (12). These subtypes can provide valuable risk stratification and prognosis estimation information beyond traditional markers.

#### 4.2.5 Prevention and treatment of heart failure and sudden cardiac death

HF is a final typical phenotype in DCM, representing a significant impairment in left ventricular (LV) systolic function. The natural history of HF in DCM includes three distinct pathways, (1) a structural and functional recovery of heart; (2) remission of symptoms and improvement/stabilization of LV contractile function; (3) progression to end-stage HF and heart transplantation/death (55). Guideline-directed medical therapy for HF is the therapeutic cornerstone in DCM, which possess definite evidence of benefits on survival (56). For end-stage refractory HF, heart transplantation is the only established surgical treatment, and implantation of a left ventricular assist device is the main mechanical circulatory support method (57), resulting in long periods of clinical stability in DCM. Cardiac resynchronization therapy (CRT) is recommended for HF patients with left ventricular ejection fraction (LVEF) ≤35%, QRS duration ≥150 ms, and left bundle branch block QRS morphology (25, 58). Despite the unremitting efforts persistently applied to improve the treatment of HF in cardiomyopathy, a broad understanding of the genetic causes of HF will promise new prospects for preventive or treatment options for HF management.

Traditionally, risk stratification for DCM heavily depends on the LVEF assessment. However, LVEF yields limited predictive value in the background of optimal treatment respondence. Nowadays, findings showed that arrhythmias may be the earliest manifestation in some subtypes of genetic cardiomyopathy, specifically in LMNA- and SCN5A-mediated cardiomyopathies (17). At present, only LMNA pathogenic variants are included in current guidelines where an implantable cardioverter defibrillator (ICD) is recommended for primary prevention of SCD in patients with LMNA mutation-causing DCM and clinical risk factors (Class IIb recommendation) (59). The fibrous tissue constitutes a substrate for VA that induces slow and heterogeneous conduction, favors reentrant circuits, and produces vulnerability to life-threatening ventricular tachyarrhythmias (60). The presence and extent of mid-wall fibrosis identified by late gadolinium enhancement (LGE)-CMR in DCM patients are significantly associated with increased risks of SCD or aborted SCD, independent of LVEF (5). Thus, for primary prevention of SCD in DCM, the risk stratification should consider LVEF, LGE, family history, presence of VT on monitoring, and genetic characterization (60). Because of an increased risk for SCD, arrhythmia surveillance is necessary to more properly employ device management, including pacemakers and ICD.

#### 4.2.6 Differentiating DCM from other inherited cardiomyopathies

Many genetic and phenotypic overlaps exist between DCM and other cardiomyopathies, such as arrhythmogenic, non-compaction, or HCM (specifically, end-stage HCM). ACM is often characterized as an inherited disease of the cardiac desmosome, featured by VA, and an increased risk of SCD (61). ACM, traditionally considered to involve the right ventricle, has also been identified to cause biventricular or left-sided illness that can present as dilated ventricle with arrhythmia (62). Atherogenic variants of ACM, both in desmosomal genes, such as PKP2, DSP, and DSG2, and in non-desmosomal genes (e.g., LMNA, SCN5A, FLNC, DES, TTN), have overlapped with DCM (62, 63). HCM is featured by LV hypertrophy, often with the predominant involvement of the interventricular septum. Termed a disease of the sarcomere, mutations in 9 genes encoding sarcomeric proteins have now been convincingly shown to cause HCM (64). Gene variants in MYH7 and MYBPC3 are the most common, each accounting for 80% of HCM patients; other genes (TNNI3, TNNT2, TPM1, ACTC1, MYL2, MYL3, and TNNC1) each account for 1%–5% of cases (65). Notably, most of these pathogenic variants can also cause DCM. Left ventricular non-compaction (LVNC) is characterized by LV dysfunction in excessive prominent trabeculations and deep intertrabecular recesses (66). Whole exome sequencing indicates that TTN, LMNA, and MYBPC3 are the most prevalent disease genes in LVNC (67).

Different mutations within the same contractile protein-encoding gene can lead to opposite functional changes in LV traits (e.g., different variants in MYH7, with distinct molecular effects, cause HCM and DCM) (44). Large overlaps in significant loci opposite effects have been observed for HCM and DCM in genome-wide association studies (68, 69). DCM risk alleles decrease LVEF, while HCM risk alleles increase LVEF. The splicing patterns in distinct regions of TTN are significantly different in LVNC compared with DCM patients with RBM20 variants, thus causing changes in TTN isoforms, which explains why some patients develop DCM while others present with an LVNC phenotype (70). Research on the heterogeneous pathogenetic effects of gene mutations is an emerging trend in DCM. Meanwhile, the prognosis consequences of specific gene variants in different cardiomyopathies must also be explored.

### 4.3 Prospects

Although the atlas of genetic mutations of DCM has made tremendous achievements in recent years, currently known gene variants only can account for less than half of the genetic causes of DCM. Furthermore, their molecular mechanisms still need to be understood. Thus more genome-wide genotyping studies in much larger cohorts of rigorously phenotyped probands and relatives are acquired to broaden our perception of the genomic basis of DCM. Some individuals do not present complete phenotypes, even with pathogenic variants, meaning they have reduced penetrance. Understanding the mechanisms for variable penetrance should also be *a priori*ty of future work. The clinical phenotype depends not only on the malignancy of gene variants but also on the influence of age, environmental insults, epigenetics, toxic factors, and a diversity of acquired diseases. Up to 20% of myocarditis patients may develop chronic inflammatory DCM (71). Beyond infections of viruses, myocarditis can be caused by a direct toxic or immune-mediated reaction to drugs, such as immune checkpoint inhibitors (ICIs) (72). There is an increased prevalence of rare variants (BAG3, LMNA, MYH7, TCAP, TNNT2, and TTN), particularly TTNtv, in adult and pediatric cancer patients with cancer therapy-induced cardiomyopathy (73). Therefore, the interaction mechanisms between gene variants and environmental triggers must be further studied.

### 4.4 Strength and limitations

To our knowledge, this is the first bibliometric analysis to depict the distribution of the main research forces, hotspots, and emerging trends in the genetics of DCM. This study has direct implications for clinical practice. Researchers can better understand research trends and identify areas of interest in this subject, thus conducting further research in this field.

However, this paper inevitably has the following limitations. Firstly, due to the limitations of operating software, only documents in the WoS database were included. Thus, the data source could have been more varied. Secondly, the uneven quality of the data collected in the studies may impair the credibility of knowledge mapping. Thirdly, some recently published essential documents do not receive enough citations, which may lead to omitting important information. Notwithstanding these caveats, this study mapped a research landscape of genetics in DCM.

## 5 Conclusion

The research landscape of genetics in DCM is continuously evolving. Deciphering the genetic expression profiles of DCM, illustrating the pathogenic mechanisms of gene mutations, establishing innovative treatments for HF and improved risk stratification regimes for SCD, uncovering the genetic overlaps between DCM and other inherited cardiomyopathies, as well as expanding genotype-phenotype knowledge are the main research hotspots and frontiers in the field of genetics of DCM.

## Data Availability

The original contributions presented in the study are included in the article/**Supplementary Material**, further inquiries can be directed to the corresponding authors.
