# Supplementary material for: Research landscape of genetics in dilated cardiomyopathy: insight from a bibliometric analysis
Source: Front Cardiovasc Med. 2024 Jul 12;11:1362551. doi: 10.3389/fcvm.2024.1362551 (PMC11272475; doi:10.3389/fcvm.2024.1362551)
Supplement: Supplementary file 1 [file Table1.docx]

Supplementary Material

# Supplementary Table 1

| **Label** | **Replace by** |
| --- | --- |
| animal models | animal model |
| arrhythmogenic right ventricular dysplasia-cardiomyopathy | arrhythmogenic right ventricular cardiomyopathy |
| arrhythmogenic right ventricular dysplasia | arrhythmogenic right ventricular cardiomyopathy |
| arrhythmogenic right ventricular cardiomyopathy/dysplasia | arrhythmogenic right ventricular cardiomyopathy |
| arrhythmogenic right ventricular | arrhythmogenic right ventricular cardiomyopathy |
| right ventricular dysplasia | arrhythmogenic right ventricular cardiomyopathy |
| atrial fibrillation (af) | atrial fibrillation |
| calcium channels - l-type | calcium channels |
| arrhythmias - cardiac | cardiac arrhythmia |
| ventricular remodeling | cardiac myosin |
| cardiac remodelling | cardiac remodeling |
| working group | cardiology working group |
| cardiovascular diseases | cardiovascular disease |
| cardiac disease | cardiovascular disease |
| muscle mri | cardiovascular magnetic resonance |
| magnetic resonance | cardiovascular magnetic resonance |
| magnetic resonance imaging | cardiovascular magnetic resonance |
| cardiac magnetic resonance imaging | cardiovascular magnetic resonance |
| cardiac magnetic resonance | cardiovascular magnetic resonance |
| chagas disease | chagas cardiomyopathy |
| clinical trial | clinical study |
| congenital myopathies | congenital myopathy |
| cardiomyopathy - dilated | dilated cardiomyopathy |
| dilated cardiomyopathy (dcm) | dilated cardiomyopathy |
| duchenne muscular dystrophy (dmd) | duchenne muscular dystrophy |
| endothelial cells | endothelial cell |
| familial dcm | familial dilated cardiomyopathy |
| genetic counselling | genetic counseling |
| genetic mutations | genetic mutation |
| genetic variation | genetic variant |
| genetic variants | genetic variant |
| heart diseases | heart disease |
| cardiac failure | heart failure |
| heart failure (hf) | heart failure |
| chronic heart failure | heart failure |
| congestive heart failure | heart failure |
| cardiac transplantation | heart transplantation |
| heart transplant | heart transplantation |
| hub genes | hub gene |
| defibrillators - implantable | implantable cardioverter defibrillator |
| implantable cardioverter-defibrillator | implantable cardioverter defibrillator |
| icd decisions | implantable cardioverter defibrillator |
| human pluripotent stem cells | induced pluripotent stem cell |
| induced pluripotent stem cells | induced pluripotent stem cell |
| inherited cardiomyopathies | inherited cardiomyopathy |
| genetic cardiomyopathy | inherited cardiomyopathy |
| genetic cardiomyopathies | inherited cardiomyopathy |
| intercalated disks | intercalated disc |
| ion channels | ion channel |
| lamin a/c gene | lamin a/c |
| lamins a/c | lamin a/c |
| lamin a | lamin a/c |
| ventricular assist device | left ventricular assist device |
| ventricular assist device | left ventricular assist device |
| noncompaction cardiomyopathy | left ventricular non-compaction cardiomyopathy |
| left ventricular noncompaction | left ventricular non-compaction cardiomyopathy |
| left ventricular non-compaction | left ventricular non-compaction cardiomyopathy |
| limb girdle muscular dystrophy | limb-girdle muscular dystrophy |
| mouse models | mouse model |
| muscular dystrophies | muscular dystrophy |
| mutations | mutation |
| neuromuscular disorders | neuromuscular disease |
| next-generation sequencing | next generation sequencing |
| pediatric dilated cardiomyopathy | peripartum cardiomyopathy |
| reverse remodelling | reverse remodeling |
| single-cell rna sequencing | rna sequencing |
| statement | scientific statement |
| signal transduc-tion | signal transduction |
| single nucleotide polymorphisms | single nucleotide polymorphism |
| stem cells | stem cell |
| sudden death | sudden cardiac death |
| transcription factors | transcription factor |
| mice | transgenic mice |
| ventricular arrhythmias | ventricular arrhythmia |
| whole-exome sequencing | whole exome sequencing |
